# Isolation and characterisation of crude oil sludge degrading bacteria

**DOI:** 10.1186/s40064-016-3617-z

**Published:** 2016-11-09

**Authors:** Linda U. Obi, Harrison I. Atagana, Rasheed A. Adeleke

**Affiliations:** 1 Department of Environmental Sciences, University of South Africa, Johannesburg, South Africa; 2 Institute for Science and Technology Education, University of South Africa, Pretoria, South Africa; 3 Microbiology and Environmental Biotechnology Research Group, Agricultural Research Council – Institute for Soil, Climate and Water, Pretoria, South Africa; 4 Unit for Environment Science and Management, North-West University (Potchefstroom Campus), Potchefstroom, South Africa

**Keywords:** Bioremediation, Crude oil sludge, Polycyclic aromatic hydrocarbon (PAHs), Composting, Bacteria, 16S rRNA, Catechol-2,3-dioxygenase

## Abstract

**Background:**

The use of microorganisms in remediating environmental contaminants such as crude oil sludge has become a promising technique owing to its economy and the fact it is environmentally friendly. Polycyclic aromatic hydrocarbons (PAHs), as the major components of oil sludge, are hydrophobic and recalcitrant. An important way of enhancing the rate of PAH desorption is to compost crude oil sludge by incorporating commercial surfactants, thereby making them available for microbial degradation. In this study, crude oil sludge was composted for 16 weeks during which surfactants were added in the form of a solution.

**Results:**

Molecular characterisation of the 16S rRNA genes indicated that the isolates obtained on a mineral salts medium belonged to different genera, including *Stenotrophmonas*, *Pseudomonas*, *Bordetella*, *Brucella*, *Bacillus*, *Achromobacter*, *Ochrobactrum*, *Advenella*, *Mycobacterium*, *Mesorhizobium*, *Klebsiella*, *Pusillimonas* and *Raoultella*. The percentage degradation rates of these isolates were estimated by measuring the absorbance of the 2,6-dichlorophenol indophenol medium. *Pseudomonas* emerged as the top degrader with an estimated percentage degradation rate of 73.7% after 7 days of incubation at 28 °C. In addition, the presence of the catabolic gene, catechol-2,3-dioxygenase was detected in the bacteria isolates as well as in evolutionary classifications based on phylogeny.

**Conclusions:**

The bacteria isolated in this study are potential agents for the bioremediation of crude oil sludge.

## Background

High demand for petroleum products all over the world has led to an increase in crude oil extraction and processing. This has resulted in an increased generation of large amounts of oily waste (Bhattacharyya and Shekdar 2003). An important example of such waste is crude oil sludge, which is a highly viscous material consisting of a mixture of oil contaminated sand (clay, silica and oxides) and the associated chemicals and water used during the refining process (Heidarzadeh et al. 2010). Crude oil sludge is mainly generated during the cleaning of storage tanks and the treatment of waste water from refineries. The constituents of crude oil sludge are toxic, mutagenic and carcinogenic and may persist in the environment for prolonged periods, posing a major threat to ecosystems (Wu et al. 2008; Ayotamuno et al. 2011; Balachandran et al. 2012; Enabulele and Obayagbona 2013). One of the major limitations of polycyclic aromatic hydrocarbons (PAHs) biodegradation is the low bioavailability of pollutants to the degrading microorganisms. The incorporation of commercially available surfactants in bioremediation techniques is thus a possible option for increasing the bioavailability of polycyclic aromatic hydrocarbons (PAHs) (Wong et al. 2004; Wyrwas et al. 2011).

The efficient disposal of crude oil sludge has become a growing concern as improper disposal results in serious environmental pollution (Loick et al. 2009). The need to remediate this contaminant has thus led to the development of certain technologies (physical and chemical) that promote the destruction, relocation, immobilisation and confinement of the oil sludge. Bioremediation is a biological approach that involves the use of microorganisms’ metabolic potential to degrade pollutants into innocuous compounds (Milic et al. 2009; Hara et al. 2013; Singh and Chandra 2014). Because most constituents of crude oil sludge are biodegradable, the use of bioremediation techniques has proven to be economical, environmentally friendly and flexible (Niti et al. 2013).

Compost bioremediation is a form of bioremediation that involves adding of composting ingredients to contaminated wastes, where the compost matures in the presence of the contaminants (Antizar-Ladislao et al. 2008). Owing to the presence of a diverse microbial populations composting of oil sludge is a simple operation and results in high treatment efficiency (Antizar-Ladislao et al. 2006; Sayara et al. 2011; Badr El-Din et al. 2014).

Several studies have reported on the catabolic abilities of microorganisms such as fungi, bacteria and algae to degrade petroleum hydrocarbons (Riser-Roberts 1992; Dean-Ross et al. 2002; Bundy et al. 2004; Wang et al. 2011; Maiti et al. 2012; Ahirwar and Dehariya 2013; Badr El-Din et al. 2014); these microorganisms possess specific enzyme systems that enable them to degrade and utilise hydrocarbons as their carbon and energy sources (Panda et al. 2013). The most important means of aerobic PAH biodegradation is the primary oxidation of the aromatic benzene ring through which molecular oxygen is incorporated by the dioxygenase enzymes to form *cis*-dihydrodiols. The intermediates of dihydrodiol dehydrogenation are metabolised to carbon dioxide and water through the catechols by the actions of catechol dioxygenases and other enzymes (Chikere et al. 2011). The present study deals with the isolation and characterisation of the bacteria that are capable of growing in and utilising crude oil sludge as their sole source of carbon and energy during compost bioremediation of crude oil sludge. The presence of catabolic enzymes such as catechol dioxygenases was subsequently ascertained using both culture dependent and culture independent methods.

## Methods

### Experimental set-up

The experimental design consisted of 15 transparent laboratory scale composting bins of about 20 cm × 40 cm in size, with lids. The sides and lids of the composters were perforated for aeration purposes. Each of the composting bins contained 1.5 kg of top soil, 300 g of crude oil sludge and 180 g of bark chips. Different proportions of soybean meal and horse manure were then added to different composters. Accordingly, 1 kg of soybean meal was added to the first five sets of composters; 500 g of soybean meal and 500 g of horse manure to the next five sets and 1 kg of horse manure to the last five sets. In addition, anionic and non-ionic surfactants were added to the compost matrix at different concentrations of 0.5 and 1.0%. The compost was set up in the pot house of Agricultural Research Council-Institute for soil, Climate and Water.

#### Sampling

Samples were collected from the composting vessels after 16 weeks of composting, transported to the laboratory in sterile plastic containers and stored at 4 °C until they were used.

#### Media

Three different media were used in this study: Bushnell Haas broth consisting of 0.2 g MgSO_4_, 0.02 g CaCl_2_, 1.0 g KHPO4, 1.0 g K_2_HPO_4_, 1.0 g NH_4_NO_3_, 0.05 g FeCl_3_ per litre of distilled water, pH 7.0; a mineral salts medium consisting of 5.0 g NaCl, 5.0 g KH_2_PO_4_, 1.0 g K_2_HPO_4_, 1.0 g (NH_4_)_2_SO_4_, 0.25 g MgSO_4_·7H_2_O, 2.0 g NaNO_3_, 0.02 g FeCl_2_·4H_2_O, 0.02 g CaCl_2_ per litre of distilled water, pH 7.2; and mineral salts agar consisting of a mineral salts medium +12 g of bacteriological agar per litre of distilled water.

### Isolation of bacteria

Bacteria were isolated by means of the enrichment culture technique (Liu et al. 2010, 2014). Two hundred and fifty millilitre Erlenmeyer flasks containing 100 ml of sterile mineral salts medium, 1.5 g of compost samples (1.5%) and 1 ml of crude oil sludge (1%) were incubated in a rotary incubator at 130 revolutions per minute (rpm) and 28 °C. The control treatment did not include added compost. An aliquot of 5 ml was sub-cultured onto a fresh mineral salts medium with 1% crude oil sludge every 21 days and incubated under the same conditions. After three transferences, a flame sterilised loop was used to streak the culture onto plates that contained mineral salts agar; these were then incubated at 28 °C for 7 days. After several sub-culturing exercises pure isolates were obtained. Pure colonies were then stored in nutrient agar slants for further characterisation.

### Identification and characterisation of oil sludge degrading bacterial isolates

#### Morphological characterisation

Pure colonies of oil sludge degrading bacteria were identified and characterised based on the results of their gram reaction tests and their morphological features when compared to *Bergey’s manual* (Garrity et al. 2005; Cerqueira et al. 2011).

### Molecular characterisation

#### Sequence analysis of the 16S rRNA gene

Colony polymerase chain reaction (PCR) was used to amplify the target 16S rRNA region of the DNA in bacterial cells. The process was performed by picking a single colony of bacteria isolates from the nutrient agar medium using the tip of a sterile pipette and placing it in 100 µl of sterile distilled water in a 1.5 ml microcentrifuge tube. The tube was incubated at between 94 and 95 °C for 10 min using a digital dry bath (Bio Rad). A volume of 2 µl was used as a DNA template for the amplification reaction. The 16S rRNA region was amplified by PCR using the forward primer, 27F (5′-AGA GTT TGA TCC TGG CTC AG-3′) and reverse primer 1492R (5′-CGG CTA CCT TGT TAC GAC TT-3′) (Liu et al. 2014). The amplification reaction was prepared using 10 µl of 2× PCR Master Mix (Thermo Scientific Phusion Flash High-Fidelity), 1 µl of each forward and reverse primer (10 µM), 2 µl of the DNA template and 6 µl of sterile distilled water resulting in a 20 µl reaction volume. The negative control was set up without genomic DNA. The amplification reaction was performed in a thermal cycler (Bio Rad T100™) as follows: one cycle at 98 °C for 10 s, followed by 34 cycles at 98 °C for one second, 53 °C for 1 min and 72 °C for 15 s. A final extension step at 72 °C for 1 min was performed for 1 cycle. The reaction was held at 4 °C until the amplicons were removed from the thermal cycler. The amplicons were then assessed by running 1% agarose gel electrophoresis and viewed in the Gel Doc imager (Bio Rad). PCR products were sent to Inqaba Biotechnological Industries for purification and sequencing. The amplified 16S rRNA gene sequences were aligned using the Bioedit and CLUSTALW software. The Basic Local Alignment Search Tool (BLAST) program of the National Centre for Biotechnology Information (NCBI) was used to search and identify the closest species. The Mothur 1.25.1 software program was then used to cluster similar sequences into OTUs (operational taxonomic units). Finally, Simpsons Index of Diversity (Simpson 1949; Bowman et al. 2012) was used to define the community structure.

Simpson’s Index of Diversity = 1 − D:

$$D = \frac{{\sum {\text{n}}\left( {{\text{n}} - 1} \right)}}{{{\text{N}}\left( {{\text{N}} - 1} \right)}}$$

where n = total number of organisms of a particular species, and N = total number of organisms of all species.

### Nucleotide sequence accession numbers

The partial 16S rRNA gene sequences in this study were deposited in the Genebank database under the accession numbers KT337506 to KT337538 and KT445946 to KT445948. A phylogenetic tree was constructed using the software, Molecular Evolutionary Genetics Analysis (MEGA) version 6.0.

### Screening of bacterial isolates for oil sludge degradability

#### Preliminary screening with oil sludge

Bacteria isolates were screened for their ability to grow in oil sludge by sub-culturing on mineral salts agar that was laced with 1.5% crude oil sludge and incubated at 30 °C for 3–7 days (Wang et al. 2011; Liu et al. 2014). Pure colonies obtained were stored in nutrient agar slants at 4 °C for further screening and characterisation.

#### Screening with 2,6-dichlorophenol indophenol

Bacterial cultures were transferred from nutrient agar slants to test tubes containing Bushnell Haas broth and incubated for 24 h at 37 °C at 180 rpm. A mixture of 0.5% (w/v) 2,6-dichlorophenol indophenol (2,6-DCPIP), 0.1% Tween 80 and 3% (v/v) crude oil sludge was then introduced into the tubes. The experiment was monitored daily for colour change from blue to colourless. The control experiment was prepared without inoculum and treatments were in duplicate (Hanson et al. 1993; Bidoia et al. 2010; Varjani et al. 2013; Ahirwar and Dehariya 2013). After 7 days of incubation at 28 °C under rotatory conditions and having observed the colour change, the liquid medium was filtered to separate the biomass. The filtrate was centrifuged at 8000 rpm for 15 min. The supernatant was then analysed at 609 nm using the ultra violet-visible (UV–VIS) spectrophotometer (Hach spectrophotometer DR 5000). The percentage of biodegradation was subsequently estimated as follows:

$$\% \;{\text{of}}\;{\text{degradation}} = 1 - \frac{{{\text{Absorbance}}\;{\text{of}}\;{\text{treated}}\;{\text{sample}} }}{{{\text{Absorbance}}\;{\text{of}}\;{\text{control}}}} \times 100$$

### Detection of catechol-2,3-dioxygenase genes in bacteria isolates

Catechol-2,3-dioxygenase is an important extradiol dioxygenase in the metabolism of aromatic rings by soil bacteria (Broderick 1999; Kasuga et al. 2007). Colony PCR was subsequently performed on the bacteria isolates, and specific forward primer C230F (5′-AAG AGG CAT GGG GGC GCA CCG GTT CGA-3′) and reverse primer C230R (5′-TCA CCA GCA AAC ACC TCG TTG CGG TTG CC-3′) (Kasuga et al. 2007; Hesham Ael et al. 2014) were used to amplify the catechol-2,3-dioxygenase genes in a 20 µl PCR reaction. The cycling conditions were as follows: one cycle at 98 °C for 10 s, followed by 34 cycles at 98 °C for one second, 55 °C for 1 min and 72 °C for 15 s. A final extension step at 72 °C for 1 min was performed for one cycle. The PCR products were then examined in 1% agarose gel electrophoresis.

## Results

### Isolation of bacteria

 After the enrichment cultivation, a total of 36 bacterial isolates were obtained from the compost samples and the control (oil sludge) (Table 1). Bacteria were also isolated from the control treatment that did not contain compost. The enrichment cultivation technique gave rise to bacteria isolates that were capable of resisting the toxicity of crude oil sludge while using it for their own carbon and energy requirements.

**Table 1:** Morphological characterisation, gram reaction test results and molecular identification of bacteria isolates from compost

Bacteria isolates ID	Shape of bacteria	Gram reaction test	Molecular identification (most likely identical taxonomic species)	Homology (%)
S2C	Bacilli	−	*Stenotrophomonas maltophilia*	99
S2E	Bacilli	−	*Stenotrophomonas maltophilia*	99
S5A	Coccobacilli	−	*Bordetella avium*	97
S5B	Coccobacilli	−	*Brucella ceti*	99
S5C	Coccobacilli	−	*Bordetella avium*	98
S8D	Bacilli	+	*Bacillus subtilis*	99
S11A	Bacilli	−	*Pseudomonas denitificans*	98
S11E	Bacilli	+	*Bacillus subtilis*	100
S11F	Bacilli	+	*Bacillus subtilis*	100
S14A	Bacilli	−	*Achromobacter xylosoxidans*	99
S14C	Bacilli	−	*Ochrobactrum anthropi*	99
S14D	Bacilli	−	*Ochrobactrum anthropi*	99
S14D1	Bacilli	−	*Stenotrophomonas maltophilia*	97
S14E	Bacilli	−	*Stenotrophomonas maltophilia*	97
SH17B	Cocci	−	*Advenella kashmirensis*	94
SH20A	Baccilli	−	*Ochrobactrum anthropi*	99
SH20B	Bacilli	−	*Achromobacter xylosoxidans*	99
SH23A	Bacilli	+	*Mycobacterium gilvum*	88
SH23B	Bacilli	−	*Ochrobactrum anthropi*	99
SH23C	Bacilli	−	*Ochrobactrum anthropi*	99
SH26A	Bacilli	−	*Pseudomonas denitrificans*	99
SH26B	Bacilli	−	*Mesorhizobium opportunistrum*	97
SH29B	Bacilli	−	*Achromobacter xylosoxidans*	99
H35A	Bacilli	−	*Achromobacter xylosoxidans*	99
H35B	Bacilli	−	*Ochrobactrum antropi*	98
H35C	Bacilli	−	*Pseudomonas denitrificans*	99
H38A	Bacilli	−	*Psedomonas denitrificans*	99
H38C	Bacilli	−	*Klebsiella varricola*	82
H41C	Bacilli	+	*Bacillus subtilis*	99
H41D	Bacilli	+	*Bacillus subtilis*	99
H41F	Bacilli	+	*Bacillus subtilis*	99
H41H	Bacilli	+	*Bacillus subtilis*	99
H44B	Bacilli	−	*Pseudomonas denitrificans*	99
Control A	Coccobacilli	−	*Pusillimonas* sp.	95
Control B	Bacilli	−	*Raoultella ornithinolytica*	99
Control D	Coccobacilli	−	*Pusillimonas* sp.	95

### Identification and characterisation of oil sludge degrading bacterial isolates

**Fig. 1:**
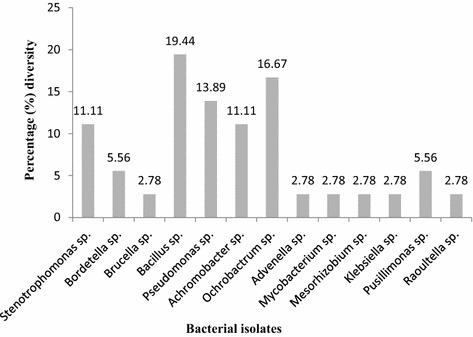
Diversity percentage (%) of the genus bacteria isolated in this study

The morphological characterisation and gram test reaction indicated the presence of 28 g-negative and 8 g-positive bacteria, of which bacilli formed 83.33% and cocobacilli and cocci represented 13.89 and 2.78% of the population respectively (Fig. 1). The phylum Proteobacteria was found to be the dominant species, representing about 77.78% of the population. Among the Proteobacteria, *γ*-proteobacteria (39.38%) and *β*-proteobacteria (35.71%) were the dominant class, while *α*-proteobacteria formed the remaining 25%. Firmicutes and actinobacteria represented 19.44 and 2.78% of the population, respectively.

### Molecular characterisation

Colony PCR of the bacteria isolates gave amplicons of about 1465 base pairs in size. The presence of white bands signified the presence of the 16S rRNA genes in the amplified bacteria isolates. The negative control (Sabax sterile water) showed the absence of the genes as no bands was seen on the 1% agarose gel electrophoresis.

The analysis of the partial sequence of the 16S rRNA showed that the bacteria isolates belonged to the genera *Stenotrophmonas*, *Pseudomonas*, *Bordetella*, *Brucella*, *Bacillus*, *Achromobacter*, *Ochrobactrum*, *Advenella*, *Mycobacterium*, *Mesorhizobium*, *Klebsiella*, *Pusillimonas* and *Raoultella*.

The sequences of the bacteria isolates were used to generate operational taxonomic unit (OTU) to enable straightforward classifications.

**Fig. 2:**
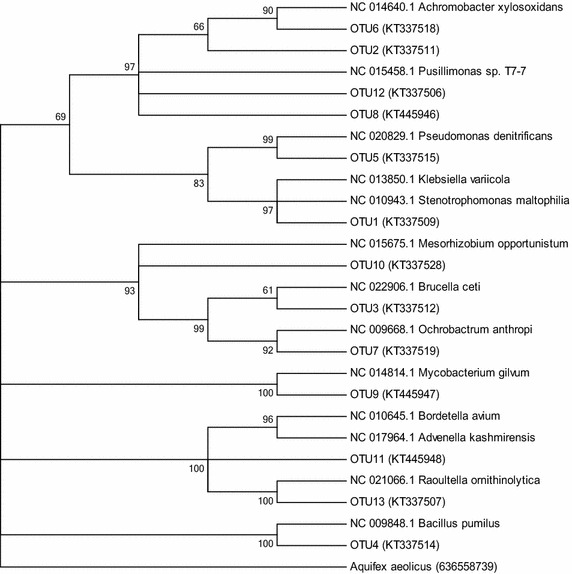
Phylogenetic analysis of the partial 16S rRNA gene sequences (1465 bp) of the bacteria isolates

The BLAST program identified species related to the nucleotides of the DNA sequences. Following the sequencing of the 16S rRNA genes, these sequences were grouped into thirteen OTUs with more than 95% similarity following the sequencing of the (Table 2; Fig. 2). Bootstrap analysis with 1000 repetitions was performed and only values higher than 50% are shown.

**Table 2:** Sequences, their OTU representatives and classifications

OTUs	No. of sequences	OTU representatives	Phylum	Class
OTU1	4	*Stenotrophomonas maltophilia*	Proteobacteria	*γ*-proteobacteria
OTU2	2	*Bordetella avium*	Proteobacteria	*β*-proteobacteria
OTU3	1	*Brucella ceti*	Proteobacteria	*β*-proteobacteria
OTU4	7	*Bacillus subtilis*	Firmicutes	Bacilli
OTU5	5	*Pseudomonas denitrificans*	Proteobacteria	*γ*-proteobacteria
OTU6	4	*Achromobacter xylosoxidans*	Proteobacteria	*β*-proteobacteria
OTU7	6	*Ochrobactrum anthropi*	Proteobacteria	*α*-proteobacteria
OTU8	1	*Advenella kashimirensis*	Proteobacteria	*β*-proteobacteria
OTU9	1	*Mycobacterium gilvum*	Actinobacteria	Actinobacteria
OTU10	1	*Mesorhizobium opportunistrum*	Proteobacteria	*α*-proteobacteria
OTU11	1	*Klebsiella varricola*	Proteobacteria	*γ*-proteobacteria
OTU12	2	*Pusillimonas* sp.	Proteobacteria	*β*-proteobacteria
OTU13	1	*Raoultella ornithinolytica*	Proteobacteria	*γ*-proteobacteria

Simpson’s Index of Diversity (1-D) was calculated as 0.9; the greater the value of 1-D, the greater the sample diversity. On the other hand, Simpson’s Index (D) was 0.1; the larger the value of D, the lower the diversity. The distribution of microorganisms was found to be heterogeneous. The most dominant group of isolates, OTU 4 at genus level was closely related to *Bacillus* which represented about 19.44% of all the isolates. This was followed by OTU 5 and OTU 7 which represented about 13.89 and 16.67% of the population, respectively (Fig. 1; Table 2).

### Screening of bacterial isolates for oil sludge degradability

The results showed that, after 7 days incubation all the bacteria isolates were able to grow on the mineral salts agar laced with crude oil sludge. This culture medium contained no carbon; the only source of carbon was the sterile crude oil sludge that was laced on the mineral salts agar plates.

**Fig. 3:**
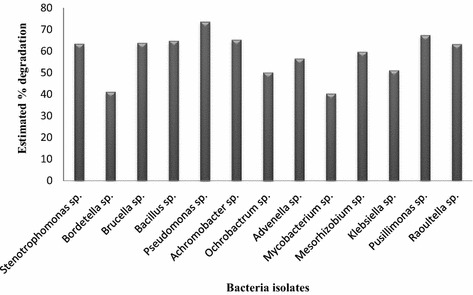
Estimated percentage degradation rate of crude oil sludge by the bacteria isolates using 2,6-DCPIP

Thirty-two of the 36 isolates showed a positive reaction to the indicator, 2,6-DCPIP. *Pseudomonas* sp. was identified as the best degrader (Fig. 3) as it was able to decolourise the 2,6-DCPIP in the shortest possible time. In addition, *Pusillimonas*, *Achromobacter* and *Bacillus* sp. were also among the top degraders.

### Detection of catechol-2,3-dioxygenae genes in bacteria isolates

The presence of the cathecol-2,3-dioxygenase (C23O) genes was detected using the PCR technique with degenerate primers. The results showed that all the isolates possessed the C23O genes.

## Discussion

The aim of this study was to isolate and characterise bacteria that are capable of using crude oil sludge for their carbon and energy requirements. This could be of further use in the bioaugmentation remediation process for crude oil sludge. According to the morphological characterisation and gram test reaction of the isolates (Table 1), composting of crude oil sludge gave rise to many bacteria isolates especially the gram-negative bacteria. Microbial degradation of oil sludge using gram-negative bacteria has been reported to improve the remediation of hydrocarbons (Zhang et al. 2011). The dominant species belong to the phylum Proteobacteria, which is comprised mainly of *β*-proteobacteria and *γ*-proteobacteria (Table 2). This result complies with the observations of Tan and Ji (2010) that these classes of bacteria possess the ability to use the nitrogen–sulphur–oxygen (NSO) fractions of crude oil sludge as their sources of nitrogen, carbon and energy.

According to our findings, organisms with PAH degrading ability belong to the genera *Stenotrophmonas, Pseudomonas, Bordetella, Brucella, Bacillus, Achromobacter, Ochrobactrum, Advenella, Mycobacterium, Mesorhizobium, Klebsiella, Pusillimonas* and *Raoultella*. This result is comparable to the findings of some studies (Hara et al. 2013; Molina et al. 2009; Mishra et al. 2014) that accentuated the abilities of most of the above-mentioned isolates to use crude oil sludge for their carbon and energy requirements. *Bacillus* and *Ochrobactrum* formed the largest population with values of 19.44 and 16.67%, respectively (Fig. 1). This could be due to their ability to proliferate more than other organisms in the presence of toxic organic material such as crude oil sludge.

Incorporating 2,6-DCPIP (an electron acceptor) in a culture medium made it possible to determine the capability of microorganisms to utilise substrate which in this study was crude oil sludge. The colour change of 2,6-DCPIP from blue (oxidised) to colourless (reduced) signified the utilisation of the crude oil sludge. This procedure was first applied in the biodegradation of oil in the method developed by Hanson et al. (1993). Among the assessed organisms, *Pseudomonas* sp. emerged as the best degrader with about a 73.7% rate of biodegradation demonstrated by its rapid decolouration of the redox indicator (Fig. 3). *Pusillimonas* sp. and *Achromobacter* sp. also exhibited their potential in degrading crude oil sludge by changing the initial colour of the 2,6-DCPIP to colourless. Some reports have classified *Pseudomonas* and *Bacillus* genera as bio-emulsifiers with the potential to increase the bioavailability of PAHs for improved biodegradation (Yu et al. 2007; Mishra et al. 2014). Many other bacteria species also have the potential to be good bioremediation agents as a result of their ability to degrade petroleum wastes and toxic organic solvents. For instance, genera such as *Ochrobactrum, Bacillus, Pseudomonas, Advenella, Achromobacter* and *Stenotrophomonas* have all been reported to play an active role in the biodegradation of crude oil sludge (Katsivela et al. 2005; Zhang et al. 2005; Veeranagouda et al. 2006; Rajaei et al. 2013; Santisi et al. 2015). These strains were able to use both aliphatic and aromatic hydrocarbons as their sole source of carbon and energy owing to their possession of the aromatic and aliphatic catabolic pathways (Rajaei et al. 2013). The inability of a few isolates to induce a colour change after 7 days of incubation did not really mean that they were unable to degrade crude oil sludge. Some of the isolates tend to be faster degraders than others, i.e. they change the colour of the indicator from blue to colourless within 7 days. Some environmental factors such as pH and temperature as well as the bioavailability of the oil sludge could be responsible for such an outcome (Karigar and Rao 2011). Based on this, a confirmatory test was also done using molecular tools to verify the presence of the catabolic genes in the bacteria isolates.

The aerobic metabolism of aromatic compounds results in the formation of three intermediates, namely catechol, protocatechuate and gentisic acid. These intermediates are further metabolised to forms in which they can be accessed by microorganisms such as simple acids and aldehydes (Mishra et al. 2014; Singh et al. 2013). The results of the assay of the cathecol-2,3-dioxygenase genes using a specific set of primers established that the bacteria isolates possessed these genes, which could be an indication that those bacteria also possess the catabolic abilities. This conforms to the findings of Mesarch et al. (2000) and Hara et al. (2013) that the presence of these genes in organisms can be an indication of their ability to degrade hydrophobic compounds. Accordingly, the presence of the catechol-2,3-dioxygenase genes in the bacteria isolates signifies the capability to degrade the crude oil sludge.

## Conclusion

The capability of pure bacteria cultures to grow in and utilise crude oil sludge as their sole source of carbon and energy provides an environmentally friendly and economical process for dealing with such sludge. In view of the toxicity of crude oil sludge to humans and the environment, the isolation of pure bacteria cultures from compost mixtures for bioaugmentation purposes could be a step in the right direction. The inclusion of commercially available surfactants in our experiment encouraged the mass transfer of PAHs to the aqueous phase, thereby making them available for microorganisms to degrade.

## Abbreviations

BLAST: Basic Local Alignment Search Tool
MEGA: molecular evolutionary genetics analysis
OTU: operational taxonomic unit
PAH: polycyclic aromatic hydrocarbon
UV–VIS: ultra violet-visible
2,6-DCPIP: 2,6-dichlorophenol indophenol
